# New Trends in Airway Management During Endoscopic Retrograde Cholangiopancreatography: A Narrative Review

**DOI:** 10.3390/jcm14165905

**Published:** 2025-08-21

**Authors:** Federica Maiellare, Fabio Sbaraglia, Miryam Del Vicario, Riccardo Fattore, Giuliano Ferrone, Monica Lucente, Alessandra Piersanti, Domenico Posa, Giorgia Spinazzola, Daniele De Padova, Caterina Malatesta, Carmela Memoli, Marco Rossi

**Affiliations:** Department of Anesthesia and Intensive Care, Fondazione Policlinico Universitario A. Gemelli IRCCS, Università Cattolica del Sacro Cuore, 00136 Rome, Italy; fabio.sbaraglia@policlinicogemelli.it (F.S.); miryam.delvicario@policlinicogemelli.it (M.D.V.); riccardofattore96@gmail.com (R.F.); giuliano.ferrone@policlinicogemelli.it (G.F.); monicalucente@gmail.com (M.L.); alessandra.piersanti@policlinicogemelli.it (A.P.); domenico.posa@guest.policlinicogemelli.it (D.P.); giorgia.spinazzola@policlinicogemelli.it (G.S.); daniele.depadova@policlinicogemelli.it (D.D.P.); caterina.malatesta@policlinicogemelli.it (C.M.); carmela.memoli@policlinicogemelli.it (C.M.); marco.rossi@policlinicogemelli.it (M.R.)

**Keywords:** airway management, ERCP, general anesthesia, sedation

## Abstract

Over time, endoscopic retrograde cholangiopancreatography (ERCP) evolved into the preferred method for both diagnosing and treating diseases of the biliary, pancreatic, and ampullary systems. Traditionally performed under “conscious” sedation, anesthesiological management during ERCP increasingly involves the use of general anesthesia (GA) due to the complexity of procedures and patient comorbidities. This narrative review aims to underscore the current absence of definitive evidence supporting a single airway management strategy during ERCP. In each section, we examine the strengths and limitations of various airway management strategies, including spontaneous breathing, endotracheal intubation, and newer techniques such as high-flow nasal oxygen (HFNO) and supraglottic airway devices (SGAs), tailored for endoscopic procedures. We explore and discuss the multifactorial determinants that influence clinical decision-making, including patient-specific risk factors, procedural complexity, resource availability, and potential complications. Any anesthesiological choice must guarantee the immobility of the patient and the versatility of the position and must be integrated with the preferences and skills of the endoscopist, the available means in the endoscopic suite, and the internal protocols. Spontaneous breathing with sedation may be appropriate for low-risk, short-duration procedures but carries risks of hypoventilation and aspiration, while GA with a device to manage airways improves procedural conditions and perioperative risks. Still, it is resource-intensive and may delay recovery. Transitions between different strategies are inherently fluid, reflecting the need for a flexible, patient-centered approach tailored to the specific clinical context. Rigorous future research is essential to establish evidence-based guidelines that enhance both safety and efficiency of airway management in this setting.

## 1. Introduction

Endoscopic retrograde cholangiopancreatography (ERCP) is a specialized diagnostic and therapeutic procedure that combines upper gastrointestinal endoscopy and x-rays to diagnose and treat problems of the duodenum, gallbladder, bile, and pancreatic ducts. Airway management during ERCP has been the object of controversy for many years [1].

Hypoxemia is one of the most frequent complications of endoscopic procedures, and it has been associated with a range of adverse clinical outcomes [2]. This risk highlights the importance of choosing an appropriate sedation and airway management strategy for each individual case.

Traditionally, gastrointestinal endoscopy has been performed with the patient under the misnamed “conscious sedation” or more properly “light sedation,” often without anesthesiological support [3]. However, as increasingly complex procedures are introduced and patients with more severe comorbidities are being scheduled, general anesthesia (GA) has gradually replaced sedation as the preferred technique. Despite the broader adoption of GA, the role of procedural analgo-sedation remains central in ERCP, and the use of standardized sedation depth scales has been proposed to better define and tailor patient care [4,5]. The literature has not demonstrated a clear superiority of one approach over the other, and no conclusive evidence has been established yet [6]. Proponents of monitored anesthesia care (MAC) with moderate to deep levels of sedation highlight its advantages, such as reduced drug administration to preserve spontaneous breathing and airway reflexes, improved patient turnover, and the potential involvement of non-anesthesiologist professionals.

Alongside an expanding range of pharmacological strategies, the development of new supraglottic airway devices and the increasing use of High Flow Nasal Oxygen (HFNO) have emerged as valuable tools to bridge the gap between spontaneous breathing and fully controlled ventilation. Conversely, GA could better control critical adverse events and prevent the discontinuation of procedures, as well as protect airways from chemical and infectious agents, but it may imply longer setup times, increased costs, and higher rates of certain perioperative complications, such as hypotension. Despite multiple attempts to provide a standardized approach to the dilemma, including an update to the guidelines in 2023 [7,8], the absence of strong evidence makes it difficult to draft a definitive clinical flowchart that includes all available options. General expert recommendations favor MAC over GA but underline the need for a comprehensive and adaptable framework that takes into account patient-specific and procedural factors in order to minimize post-procedural complications. Current endoscopic techniques indeed blur the lines of surgery and expose patients to a wide variety of adverse events, such as the risk of aspiration, hypothermia, fluid and electrolyte imbalances, pain, and complications caused by any sudden movement. Longer procedures are not easy to manage in MAC, and a “patient first” approach rather than a “technique first” mindset will recognize this for the sake of patient well-being.

This clinical review aims to explore and synthesize the evidence on airway management, highlighting the strengths and limitations of each strategy. We performed a comprehensive search of PubMed/MEDLINE, Embase, and Google Scholar databases, without temporal limits, in order to capture both historical background and the most recent evidence. Keywords and MeSH terms related to ERCP, airway management, sedation, general anesthesia, high-flow nasal oxygen, and supraglottic airway devices were combined. Priority was given to English-language prospective randomized controlled trials, systematic reviews, meta-analyses, consensus guidelines, and large observational studies; however, pertinent case series, retrospective studies, and feasibility studies were also included to reflect innovations in practice. In Table 1, we summarize relevant articles of each topic addressed in this review.

**Figure 1:**
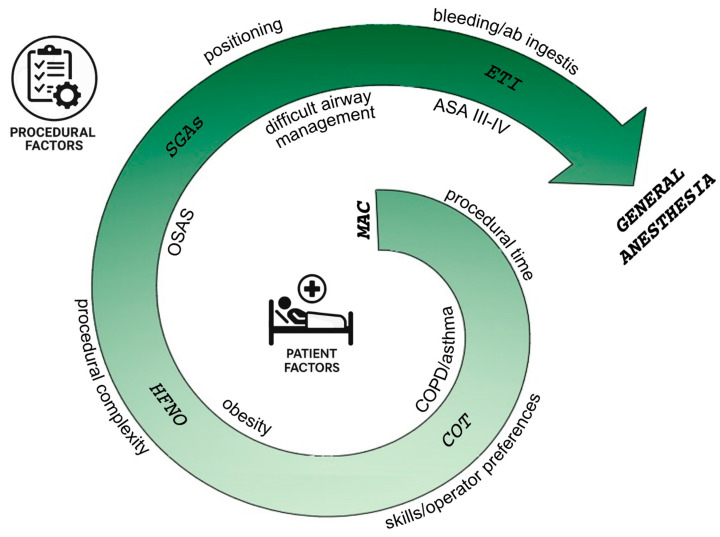
Conceptual framework for ERCP airway management. (MAC: monitored anesthesia care; COPD: chronic obstructive pulmonary disease; COT: conventional oxygen therapy; HFNO: high flow nasal oxygen; OSAS: obstructive sleep apnea syndrome; SGAs: supraglottic devices; ETI: endotracheal intubation).

Finally, we propose a visual abstract that provides a conceptual framework for driving anesthesiological choices during ERCP, illustrating the progression from monitored anesthesia care to general anesthesia. It emphasizes that airway management decisions are not dictated by a fixed algorithm but result from a comprehensive evaluation by the clinician, who must integrate both procedural complexities and individual patient characteristics. Transitions between different strategies are inherently fluid, reflecting the need for a flexible, patient-centered approach tailored to the clinical context (Figure 1).

## 2. Conventional Oxygen Therapy

Conventional oxygen therapy (COT), commonly administered via nasal cannula, remains the first-line strategy for supporting spontaneous ventilation. Maintaining spontaneous breathing during gastrointestinal endoscopic procedures represents a challenge, particularly for complex cases, such as ERCP. Careful patient selection, appropriate anesthetic agent choice, vigilant monitoring, and readiness to manage potential complications are essential for successful outcomes. 

While light sedation maintains respiratory drive, it is often insufficient for prolonged or complex procedures [16,17]. Moderate-to-deep sedation is crucial for patient compliance and procedural success, but it carries the risk of respiratory depression, potentially leading to apnea, hypopnea, and severe desaturation, requiring advanced airway management skills. Moreover, sedation may impair the gag reflex and predispose patients to aspiration of gastric content [18].

Studies have demonstrated a positive correlation between sedation depth and the incidence of aspiration pneumonia [19,20].

Adverse events, including minor respiratory incidents, occur in approximately 28% of spontaneously breathing patients under deep sedation with propofol [21]. These incidents, primarily represented by moderate desaturations, can typically be solved by the anesthesiologist without procedural interruption, using maneuvers such as jaw thrust or chin lift.

Currently, there appears to be no clear correlation between transient and moderate episodes of apnea, hypercapnia, and hypoxemia and worse postoperative outcomes for patients, such as prolonged recovery room stay, premature cessation of the procedure, unplanned intensive care admission, respiratory complications, or cardiovascular events [22].

Before undergoing ERCP, all patients should be evaluated by an anesthesiologist, who must carefully consider anatomical characteristics, comorbidities, anxiety level, the need for analgesia, as well as the procedure’s complexity and duration, and the expertise of the operators.

Risk factors associated with respiratory complications include American Society of Anesthesiologists (ASA) physical status greater than 3, body mass index (BMI) over 30 kg/m^2^, presence of comorbidities such as Obstructive Sleep Apnea Syndrome (OSAS), advanced age, Mallampati score of 3–4, and heavy alcohol use [14]. A retrospective study provides a realistic reflection of endoscopic practice in the United States and highlights the significance of the ASA physical status classification as a predictor of these complications. The authors investigated immediate adverse events requiring an unplanned intervention and demonstrated a clear positive association between a higher ASA class and periprocedural morbidity [23].

Airway assessment is essential for identifying prognostic criteria for potential difficult airways, guiding the choice between spontaneous breathing sedation and airway management through the use of supraglottic devices or endotracheal intubation. While ASA class and age are not the sole determinants, comprehensive patient evaluation remains crucial. Asthmatic and chronic obstructive pulmonary disease (COPD) patients, with hypersensitive and hyperreactive airways, may benefit from maintaining spontaneous breathing as it preserves the patient’s intrinsic respiratory drive, avoids positive pressure ventilation that can exacerbate dynamic hyperinflation and barotrauma, and reduces airway manipulation.

Although low-flow oxygen supplementation via nasal cannulae is a common practice and has been shown to reduce episodes of hypoxemia—especially in elderly and cardiopathic patients [24]—caution remains necessary when administering oxygen to chronically hypercapnic patients, as it may suppress their respiratory drive. In these patients, excessive oxygen concentration can reduce inspiratory effort; therefore, it is a reasonable com-promise to deliver oxygen at the lowest effective flow to maintain adequate peripheral saturation between 88% and 92%.

Additionally, patients with a high BMI and/or at an increased risk of OSAS should be screened using the STOP-BANG questionnaire to stratify their risk [25] and should avoid undergoing ERCP while breathing spontaneously without specific respiratory support [26].

Additionally, the use of capnography is recommended, especially in patients at risk of having apneas, as it provides a direct indicator of ventilatory activity, thus identifying ventilatory depression before hypoxemia occurs [27]. The reliability and safety of end-tidal (Et) capnography during ERCP and other complex endoscopic procedures remain poorly supported by evidence; moreover, it is not widely available. A key limitation lies in the technical feasibility of accurate EtCO_2_ monitoring, which is often hindered by procedural field interferences such as saliva, secretions, and mechanical obstructions. Recently, dedicated endoscopic capnometers utilizing the mainstream technique have been developed to address these challenges [28]. Even though there is not a clear link between a transient hypoxemia episode and serious cardiopulmonary events, such as cardiac arrhythmias or myocardial ischemia, integrating capnography into monitoring protocols for endoscopic procedures does not improve patient safety when moderate sedation is administered, but it does when the sedation is deeper [29].

The expected duration and complexity of the ERCP are the other variables that guide the choice of the anesthesiological technique and the most appropriate monitoring. The risk of procedural complications is linearly associated with its duration, but severe adverse events are generally rare [30,31]. Low-complexity and short-duration procedures (Levels I and II according to the American Society for Gastroenterology Endoscopy—ASGE) are ideally conducted with spontaneous breathing, while higher-complexity or longer procedures (ASGE Levels III and IV) warrant airway protection measures [32] (Table 2).

Special attention is required for procedures involving endoscopic placement of trans-luminal drains, ERCPs in obstructed patients, and sphincterotomies.

Finally, the choice of spontaneous breathing during ERCP may impact procedural costs and operating unit efficiency, with avoidance of orotracheal intubation leading to reduced procedure times and improved turnover [33].

The selection of sedative agents for ERCP remains a topic of debate, as the literature lacks a definitive consensus [9]. Pharmacological strategies should aim to avoid suppression of the respiratory drive while providing adequate sedation depth. Propofol, despite its widespread use due to rapid onset and recovery, can lead to dose-dependent respiratory depression, particularly in elderly or high-risk patients. To mitigate this, it is frequently combined with dexmedetomidine, which exerts sedative and sympatholytic effects without significant respiratory compromise. This agent may also be administered as a sole sedative, offering light, cooperative sedation with an analgesic component, albeit with a slower onset and a potential risk of bradycardia. The use of drug combinations is becoming increasingly common, as it allows for dose reduction and, consequently, fewer side effects [10]. These benefits can be attributed to the pharmacological synergy between agents, which optimizes sedation, analgesia, and cardiorespiratory stability. Furthermore, the improved safety and patient satisfaction associated with these regimens reinforce their clinical value—particularly in the context of ERCP, where minimizing risk while ensuring patient comfort is essential. Ketamine offers the advantage of preserving airway reflexes and ventilatory drive, and when used in low doses as an adjunct, it enhances analgesia and reduces the requirement for other sedatives. Benzodiazepines, such as midazolam, are still occasionally used for anxiolysis, though their prolonged half-life and potential for respiratory depression limit their use in prolonged procedures. Remimazolam, a new ultrashort-acting benzodiazepine receptor agonist, has been recently proposed as an alternative to propofol due to its hemodynamic advantages, but results from ERCP are still inconclusive [34]. Opioids, particularly short-acting agents like remifentanil, can improve tolerance of endoscopy, but must be titrated carefully due to their synergistic depressant effects when combined with other sedatives. However, literature has not shown significant differences in adverse event rates between studies comparing different pharmacological approaches [35].

The pharmacological plan must be dynamically adjusted throughout the procedure based on the patient’s response, depth of sedation, and airway patency, with continuous monitoring playing a pivotal role in ensuring safety.

A prospective, randomized study has shown fewer episodes of hypotension and the need for airway interventions when a single loading dose of dexmedetomidine is combined with propofol for deep sedation during ERCP in elderly patients [36]. Opioid adjuncts may not impact sedation depth or recovery, but they can enhance post-procedural patient comfort [37]. Consequently, anesthesiologists must tailor their choices to individual patient needs, considering each drug’s pharmacokinetic and pharmacodynamic profiles, as well as their personal preferences.

## 3. High-Flow Nasal Oxygen

High-flow nasal oxygen (HFNO) has emerged as a valuable noninvasive respiratory support, offering an alternative to conventional oxygenation methods, particularly during procedures, such as ERCP, where maintaining oxygenation and minimizing airway interventions are crucial. HFNO delivers heated, humidified, and precisely blended air-oxygen mixtures at high flow rates of up to 60 L/min through a specialized nasal cannula. This approach confers multiple physiological benefits: it generates a variable positive airway pressure that creates a mild positive end-expiratory pressure (PEEP) effect, reduces anatomical dead space through CO_2_ washout, and significantly decreases the work of breathing. Together, these advantages have led to the increasing adoption of HFNO in perioperative settings and procedural sedation, where it has demonstrated a reduced risk of hypoxemia and an enhanced ventilatory efficiency compared to low-flow nasal cannula oxygen [38].

The application of HFNO in the endoscopy unit has shown potential to decrease the reliance on GA for ERCP procedures, contributing to shorter periprocedural times and improved oxygenation parameters [11].

Despite the heterogeneity of the samples included in the various studies, several reviews, and meta-analyses about the use of HFNO in the context of sedation, not limited to the endoscopic setting, support the hypothesis that high flows reduce the incidence of hypoxia, increase the minimum oxygen saturation, and decrease the need for airway maneuvers [11,39]. Furthermore, in patients without COPD, HFNO did not significantly increase the risk of adverse events related to CO_2_ retention or blood pressure drops, compared to standard low-flow nasal cannula. On the contrary, high levels of supplementary oxygen could worsen hypercarbia in COPD patients because of changes in hypoxic pulmonary vasoconstriction, physiologic dead space, and reduction of hypoxemic respiratory drive [40].

When focusing on obese and high-risk comorbid patients, the efficacy of HFNO in preventing hypoxemia along with its impact on hypercarbia incidence remains controversial.

Zhang et al., in a systematic review and meta-analysis of 569 high-risk hypoxemia patients, found no significant differences between HFNO and conventional oxygen therapy in preventing oxygen desaturation below 90% or in reducing the need for airway interventions. These findings may be explained by the fact that higher fractions of oxygen are unlikely to improve hypoxia caused by sedation-induced hypoventilation or shunt, and also because these patients often require higher positive airway pressures to counteract hypoventilation. However, mouth opening during the endoscopic procedure allows gas to escape, making it difficult to maintain adequate airway pressure [41].

Conversely, Lee et al. support the theory that HFNO, in a similar and broader population of 1090 patients, provides a clinically beneficial approach to safeguard oxygenation, contributing to safer procedural sedation in gastroenterology settings [42].

The ODEPHI trial corroborates the use of HFNO in high-risk hypoxemic patients, showing that this technique could decrease the need for airway interventions and adverse events due to hypoventilation, despite the lack of data about end-tidal CO_2_ [43].

The gradual accumulation of CO_2_, due to both impaired exhalation from hypoventilation and intestinal CO_2_ insufflation, can lead to cardiovascular complications, highlighting the need for careful monitoring during the procedure [44].

Another consideration is the potential risk of increased gastric insufflation and aspiration associated with higher positive airway pressure. While this does not appear to be a concern in healthy individuals breathing spontaneously, it remains unclear whether it poses a risk in sedated patients [45].

In conclusion, HFNO provides a valuable tool for improving patient safety and comfort during ERCP, but its application requires careful consideration of patient-specific factors and a clear understanding of its potential advantages and disadvantages.

## 4. Supraglottic Devices

A supraglottic device is broadly defined as including all instruments that can be used to ventilate a patient without positioning an endotracheal tube, thus reducing the intrinsic risks of positioning a device across the vocal cords, including but not limited to edema, laceration, ulceration, cartilaginous trauma, dysphonia, and dysphagia. Furthermore, supraglottic devices eliminate the need for laryngoscopy and muscle relaxants while still providing an adequate and efficient means of ventilation in patients under GA, and their placement is easier and quicker when compared to endotracheal intubation [46,47]. Keeping in consideration that gastroenterologists have experienced significant advantages with the use of deep sedation, as it enables the execution of complex procedures with notable ease, supraglottic devices could be an excellent compromise for airway management in patients undergoing GA for endoscopic procedures. Compared to MAC, they reduce the risk of respiratory depression, improving working conditions for the endoscopist, without excessively affecting the duration of the procedure, the recovery period, and the logistic aspects [6,48].

**Figure 2:**
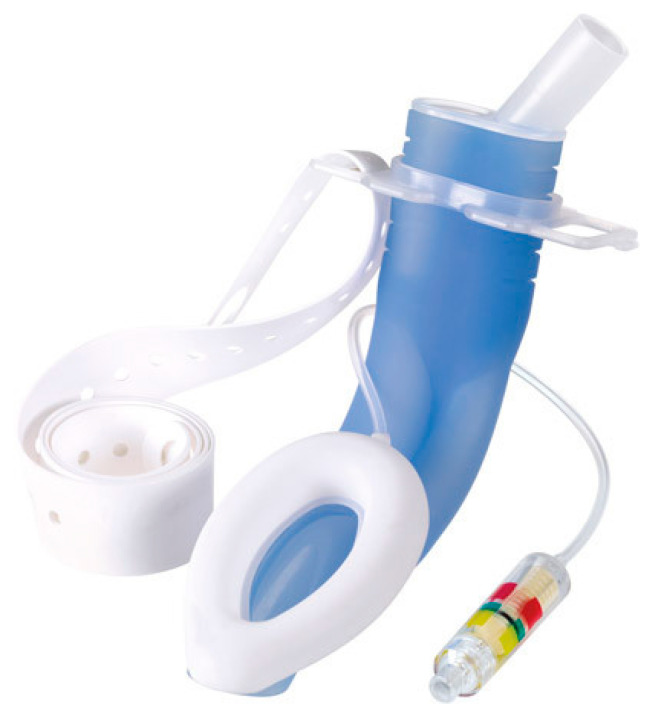
LMA^®^ Gastro™ Airway. (Image obtained by Teleflex, with written permission).

The traditional Laryngeal Mask Airway (LMA) is a common device, fully embraced by anesthesiology practitioners in and out of the operating room. One of its main limitations is the mechanical bulk, which restricts access to the endoscopic instrument. To allow upper gastrointestinal endoscopic procedures, a modified laryngeal mask with a dedicated esophageal lumen, the LMA^®^ Gastro^TM^ Airway (Teleflex^®^ Medical, Athlone, Ireland), has already been validated in many studies [12] (Figure 2). It combines the advantages of the laryngeal mask with a rate of periprocedural adverse events comparable to those occurring in patients who have undergone endotracheal intubation for ERCP. Additionally, the LMA Gastro has been observed to be a feasible alternative to endotracheal intubation, even in more complex patients, such as high-risk and pediatric patients [13,49,50].

Further consideration involves airway management in relation to patient positioning. Polese et al. explore how patient positioning influences procedural efficacy, safety, and outcomes during therapeutic endoscopic procedures. The authors emphasize that the choice of the prone position during ERCP facilitates optimal endoscope handling and procedural access. This decision should be carefully tailored to the procedural requirements, the operator preference, and the individual patient’s clinical profile. From an anesthesiological perspective, patient positioning significantly affects not only airway patency and aspiration risk but also hemodynamic and respiratory dynamics [51]. Supraglottic airway devices can be effectively used in both the prone and left lateral positions, providing an additional benefit [52].

Similarly, the gastro-laryngeal tube has been demonstrated to be a proven enhancement in the occurrence of adverse events. It allows the endoscopist and the anesthesiologist to share the entrance to the upper airway, having two separate pharyngeal ports for the endoscope and for ventilation. Although this device has shown an improvement in stress response when compared to endotracheal intubation [53], it did not demonstrate superiority in terms of the occurrence of sore throat, dysphagia, and mucosal trauma [54].

A key concern for anesthesiologists using supraglottic airway devices is the risk of an inadequate seal, which may lead to aspiration, as well as the possibility of compromised ventilation due to malposition or displacement during the procedure. To reduce these risks, a deep level of sedation is often required; however, this may affect cardiovascular stability, particularly in elderly patients or those with significant comorbidities. Nonetheless, such complications appear to be infrequent when the airway management plan is carefully individualized and developed in close collaboration with the endoscopist, based on patient characteristics, the indication for ERCP (therapeutic vs. diagnostic), and the anticipated procedure duration. When properly positioned—and, in the case of the LMA^®^ Gastro™, with appropriate cuff inflation—these devices generally provide sufficient protection against aspiration [55].

Supraglottic devices have emerged as an optimal choice for endoscopic procedures of the gastrointestinal tract, especially ERCP, as they can reduce the risk of hypoventilation, procedure-related adverse events, and recovery time, while still satisfying the needs of the endoscopist.

## 5. Endotracheal Intubation

According to the 2023 Consensus Statement, GA with tracheal intubation should be preferred in procedures with a high risk of complications, such as aspiration or massive bleeding, endoscopic ultrasound-guided transluminal access and drainage, ERCP performed in the setting of concurrent gastric outlet obstruction, prolonged cases characterized by high procedural complexity, and massive post-sphincterotomy bleeding [7].

From the perspective of personalized medicine, additional factors often considered secondary in traditional protocols, can significantly influence the anesthesiological management strategies. Tracheal intubation is usually performed with the patient in the supine position. However, as ERCP is often conducted with the patient in the semi-prone position on the endoscopy table, it is necessary to account not only for a longer periprocedural time but also for the need for a greater number of human and instrumental resources. Turning an intubated patient from supine to prone position is inherently complex and carries a degree of risk, necessitating the coordinated involvement of up to five healthcare professionals, including the anesthesiologist, endoscopist, nursing staff, and occasionally radiologists, to ensure safe and effective patient positioning [56].

Despite the recognized risks associated with the transition from the supine to the prone position in patients intubated under GA, recent evidence provides valuable insights. Xiang et al., in a randomized controlled trial, suggested that patients who were extubated while remaining in the prone position experienced statistically significant reduction in extubation time compared to patients undergoing ERCP in the supine position (13.72 (2.18) min vs. 6.99 (1.51) min, *p*  <  0.001). Similar results were found for endoscopy suit exit and discharge from the recovery room [57].

Furthermore, endotracheal intubation provides stable, reliable airway access for ventilation, and the cuffed endotracheal tube protects the airways from materials such as gastric contents or blood that could be aspirated during ERCP due to the shared airway [58].

Recent studies have explored the impact of anesthesia and airway management choices on patient and procedural outcomes, as well as efficiency, during ERCP. Althoff et al. retrospectively found that GA, when not clearly indicated, was associated with increased adverse discharge outcomes (an 8.6% risk increase compared to sedation, 95% confidence interval, 4.5–12.6%; *p* < 0.001), including mortality and prolonged hospital stays, potentially due to intraoperative hypotension [15]. However, this study acknowledged that patients undergoing GA were usually more critically ill and underwent longer, more complex procedures.

While effective airway control protects against respiratory events such as hypoxemia, GA may increase hypotension risk [59,60,61,62]. Although endotracheal intubation is often assumed to prevent pneumonia, its protective effect has not been consistently demonstrated in the literature [14]. On the contrary, some studies suggest that MAC or even no sedation might have a lower pneumonia incidence [22,63].

GA has demonstrated a procedural success rate comparable to MAC. Nevertheless, although adequate sedation is crucial for uninterrupted procedures, tracheal intubation may prolong anesthesia induction time, subsequently impacting endoscopy room turnover. However, the impact of anesthesia technique on overall endoscopy suite efficiency remains a subject of ongoing investigations. A study analyzing various endoscopic major procedures, including ERCP, found that tracheal intubation negatively affected procedural time, non-procedural time, and costs, while reducing throughput [33]. Additionally, this study emphasized that patient selection for intubation by anesthesiologists likely contributed to the observed low complication rate.

A few alternative approaches have been proposed to maximize the advantages of GA with tracheal intubation while minimizing its drawbacks. Barakat et al. investigated endoscopist-facilitated intubation in the prone position, reporting benefits such as reduced anesthesia duration, lower patient discomfort, and a decreased incidence of staff-related musculo-skeletal injuries [64]. Wu et al. introduced a “muscle relaxant-free” GA approach administering propofol and short-acting opioids. This approach allowed for successful tracheal intubation and yielded shorter procedure times compared to conventional GA methods [60].

## 6. Discussion

Despite advances in anesthesia and airway management, there is still no definitive evidence favoring one anesthetic technique over another for ERCP.

Improvements in pharmacology and monitoring technologies have expanded the feasibility of delivering a continuum of anesthetic techniques across various clinical settings—from basic monitored anesthesia care to deep sedation and full general anesthesia. The selection of the appropriate anesthesiological approach should be individualized, based on a comprehensive preprocedural assessment of each. At every stage along this continuum, airway management must be tailored with precision, such as a custom-fitted strategy, to ensure both safety and procedural efficacy.

Emerging tools such as modified SGAs and HFNO are expanding the range of options for managing the airway in sedated patients without resorting to tracheal intubation. These innovations offer promising alternatives, especially in selected clinical scenarios.

Anesthesiologists face the complex challenge of minimizing respiratory complications, preventing premature procedure interruptions, ensuring patient well-being, and simultaneously promoting timely recovery and discharge—all while maintaining the highest standards of patient safety and procedural efficiency.

These dual objectives are not only essential to optimizing patient care but may also represent an opportunity to challenge and transcend longstanding cultural barriers, rather than being regarded as impediments to innovation. It is crucial to avoid rationalizing professional inertia by appealing to tradition. Instead, the scientific community should actively encourage progress by promoting the integration of evidence-based advancements into clinical practice.

Unfortunately, currently available data about the actual incidence and safety profile of sedation practices remain inadequate and fragmented. Many old studies fail to account for recent technological advances and evolving clinical practices. Moreover, adverse events are chronically underestimated in the literature, often due to fears of legal repercussions and institutional reluctance to expose complications. Prospective data from robust, well-designed studies are still scarce, further highlighting the pressing need for updated evidence to guide clinical decisions in this rapidly changing field.

Furthermore, our narrative review has inherent limitations, as the lack of a systematic and standardized methodology, as well as formal assessment of evidence quality, limits the generalizability of the results and prevents definitive conclusions.

Pharmacological anesthetic protocols should ideally be tailored to the individual patient, based on a thorough assessment using a comprehensive multimodal approach. At a minimum, this approach should consider four critical parameters: the collective expertise and technical skills of the multidisciplinary team, the patient’s baseline condition and comorbidities, the expected duration and invasiveness of the procedure, and logistical factors such as equipment availability, the scope of medical and economic resources, and institutional preparedness for managing potential adverse events. For the same reasons, airway management strategies should likewise reflect these considerations, placing particular emphasis on provider competence and training. 

In many countries, it is increasingly common for sedation during endoscopic procedures to be administered by non-anesthesiologist personnel, such as endoscopists, nurses, or general physicians without formal anesthesia training. This model of care has primarily focused on the administration of propofol as a single agent, with promising reports suggesting both satisfactory safety outcomes and potential cost savings. However, the limited airway management expertise among these providers inevitably limits their ability to guarantee optimal patient care in more complex scenarios. It is conceivable that in the near future, the use of HFNO could be safely extended to non-anesthesiologists to enhance oxygenation strategies during sedation. Nevertheless, as of now, there is no published evidence supporting its efficacy or safety in this specific context [65].

## 7. Conclusions

No definitive anesthetic strategy has yet proven superior to enhance patient safety and procedural efficiency for ERCP, emphasizing the importance of an individualized approach that integrates patient characteristics, procedural demands, and provider expertise. Emerging tools, such as modified supraglottic devices and high-flow nasal oxygen, broaden the options for maintaining spontaneous breathing, although their optimal role remains to be established. Our review aims to update and guide anesthesiological choices, without including a comment on the technical aspects of the endoscopic procedure itself. To shape future practice, high-quality randomized controlled trials in homogeneous patient populations are needed to validate current strategies and refine sedation and airway management approaches.

## Figures and Tables

**Table 1:** Review relevant articles of each section. ERCP: endoscopic retrograde cholangiopancreatography. Pro-Dex: Propofol and Dexmedetomidine. Pro: propofol. Keto-Fol: Ketamine and Propofol. Pro-Mid Propofol and Midazolam. RCTs: randomized controlled trials. HFNO: high-flow nasal oxygen. LMA: Laryngeal Mask Airway. MAC: monitored anesthesia care. GA: general anesthesia.

Study (Ref), Year	Design	Main Topic	Population Studied	Outcomes	Findings
Zhang N et al. [9], 2024	Observational retrospective study	Pharmacological strategy	600 patients undergoing ERCP divided into four groups: - Pro-Dex group - Pro-group - Keto-Fol group - Pro-Mid group	Hemodynamic parameters, sedation level, recovery time, and procedure-related complications.	Pro-Dex protocol offers superior sedation quality, faster recovery, and fewer complications compared to other protocols during ERCP. No significant differences in the incidence of ERCP-related adverse events, hypotension, or bradycardia among the four groups.
Liu Y et al. [10], 2025	Meta-analysis	Pharmacological strategy	42 RCTs comparing patients undergoing ERCP with various drug combinations.	Procedure time, patient satisfaction, SpO_2_, incidence of SpO_2_ < 90%, and adverse events.	Combination approaches—particularly propofol with oxycodone or dexmedetomidine plus fentanyl—appear to offer an optimal balance of procedural efficiency, patient satisfaction, and a safer oxygenation profile.
Gamal et al. [11], 2022	Meta-analysis	HFNO	3 RCTs with 390 patients: - 196 HFNO - 194 low flow oxygen	Incidence of hypoxia, lowest SpO_2_, adverse events.	HFNO reduced the incidence of hypoxia in patients undergoing ERCP, provided a higher mean lowest oxygen saturation, and a lower need for airway interventions.
Hagan KB et al. [12], 2020	Prospective observational study	Supraglottic devices	30 patients undergoing ERCP with an LMA^®^ Gastro™ placement	Number of attempts and time to successful SGA placement, vital signs, SpO_2_, median end-tidal CO_2_, practitioner satisfaction, and any complications.	LMA^®^ Gastro™ is a safe alternative airway for ERCP, with high placement success (96–97%), excellent procedural completion rates (93–98%), maintained oxygenation (SpO_2_ ≥ 95%), median end-tidal CO_2_ of 35 mmHg, high practitioner satisfaction, and only minor, transient postoperative complications.
Uysal H et al. [13], 2021	Randomized controlled trial	Supraglottic devices	83 patients undergoing ERCP divided into two groups: - LMA^®^ Gastro™ group - gastrolaryngeal tube	Oropharyngeal leak pressure and supraglottic devices-related adverse events.	Oropharyngeal leak pressure and complication rate were lower in the LMA group.
Dhaliwal A et al. [14], 2021	Meta-analysis	MAC vs. GA with endotracheal intubation	21 studies with a total of 11,592 patients undergoing ERCP under MAC or GA.	Adverse events, duration of the procedure, recovery time, ERCP cannulation rates, and conversion rate of MAC to GA.	No statistically significant differences between the two groups. The mean duration of the procedure was longer in the MAC group, but the mean recovery time was shorter.
Althoff FC et al. [15], 2021	Observational retrospective study	MAC vs. GA with endotracheal intubation	17,538 patients undergoing ERCP	Adverse discharge (in-hospital mortality or new discharge to a nursing facility), intraoperative adverse events, 30- and 90-day mortality, length of stay, hospital charges, postoperative acute kidney injury and pneumonia within 30 days.	Sedation was associated with reduced adverse discharge, intraoperative hypotension, and lower length of stay.

**Table 2:** American Society for Gastroenterology Endoscopy (ASGE) classification for ERCP procedures complexity.

ASGE Levels	Procedures	Estimate Duration
I Diagnostic ERCP or simple therapeutic maneuvers	Deep cannulation of the duct of interest, main papilla, or sampling; biliary stent removal or exchange.	<30 min
II Standard therapeutic interventions with moderate complexity	Biliary stone extraction ≤ 10 mm; treatment of biliary leaks; treatment of extrahepatic strictures (benign or malignant); placement of prophylactic pancreatic stents.	30–60 min
III Advanced therapeutic procedures or multiple interventions	Biliary stone extraction ≥ 10 mm; minor papilla cannulation in divisum and therapy; removal of internally migrated biliary stents; intraductal imaging, biopsy, or fine-needle aspiration; management of acute or recurrent pancreatitis; treatment of pancreatic strictures; removal of pancreatic stones that are mobile and ≤5 mm; treatment of hilar tumors; treatment of benign biliary strictures, hilum, and above; management of suspected sphincter of Oddi dysfunction (with or without manometry).	>60 min
IV Highly complex or high-risk interventions	Removal of internally migrated pancreatic stents; removal of pancreatic stones that are impacted and/or ≥5 mm; removal of intrahepatic stones; pseudocyst drainage or necrosectomy; ampullectomy; ERCP after a Whipple procedure or Roux-en-Y bariatric surgery.	>60 min

## Data Availability

Not applicable.

## Abbreviations

ASA	American Society of Anesthesiologist
ASGE	American Society for Gastroenterology Endoscopy
BMI	body mass index
COPD	chronic obstructive pulmonary disease
COT	conventional oxygen therapy
ERCP	endoscopic retrograde cholangiopancreatography
Et	end-tidal
ETI	endotracheal intubation
GA	general anesthesia
HFNO	high flow nasal oxygen
LMA	laryngeal mask airway
MAC	monitored anesthesia care
OSAS	obstructive sleep apnea syndrome
PEEP	positive end-expiratory pressure
SGAs	supraglottic devices
